# Reimagining Black maternal health narratives: Embracing a Vitality Framework for joy, liberation, and healing

**DOI:** 10.1371/journal.pgph.0004703

**Published:** 2025-07-18

**Authors:** Ijeoma Nnodim Opara, Yasmine M. Elmi

**Affiliations:** 1 Departments of Internal Medicine and Pediatrics, Wayne State University, Detroit, Michigan, United States of America; 2 Faculty of Medicine and Health Sciences, McGill University, Montreal, Québec, Canada; University of Augsburg / Ludwig Maximillian University of Munich, GERMANY

## Abstract

Black maternal health narratives have long been dominated by crisis-focused frameworks that emphasize morbidity and mortality, often reinforcing narratives of vulnerability, pathology, and systemic failure. While these statistics are crucial in identifying disparities, they obscure the full spectrum of Black maternal experiences, erasing stories of joy, resilience, and thriving. This essay argues for a shift toward a vitality framework—one that reimagines Black maternal health beyond survival to center joy, liberation, and healing. Drawing from historical and contemporary contexts, we explore how systemic factors—including racism, sexism, and colonialism—have shaped dominant narratives, contributing to the weathering of Black bodies. We introduce ethering as a framework that transforms these harmful effects by reclaiming Black joy as an essential component of maternal health. Grounded in principles of reproductive justice, Black feminist thought, and Ubuntu, this approach moves beyond deficit-based models to highlight the collective power of Black communities in shaping new possibilities for maternal well-being. By integrating insights from public health, advocacy, and cultural expression, this essay proposes a reorientation of Black maternal health discourse toward holistic, affirming, and future-building strategies that recognize Black motherhood as a site of power, agency, and transformation.

## Introduction: The current narratives on Black maternal health

Often a person’s first thoughts or instincts when considering the experiences of a Black pregnant woman or birthing person is filled with the current narrative both within academic research, and mainstream media which shows that being Black and pregnant is dangerous and at times deadly [1–3]. Of all high-income or high-debt countries the US has the worst statistics in maternal & infant health despite spending the most with an annual healthcare expenditure of $4.5 trillion or $13,493 per person [4]. The maternal mortality ratio from pregnancy-related complications for Black women is over four times that of a white women, and in certain states like New York it’s up to 12 times higher [5]. Black women are also said to experience higher rates of severe maternal physical and mental morbidity including inadequate prenatal and postpartum care [6,7]. This narrow narrative of poor health outcomes for Black women and birthing people lends itself to explanations rooted in the default **STORYLINE** of victim-blaming, or preferably survivor-blaming, as it regards health disparities that ignore the political, historical, structural, and systemic contexts which create the conditions rendering Black women vulnerable to experiencing those poor health outcomes.

However, this storyline achieves five things:

1. This **storyline** obscures the intersectional role played by white supremacism, colonialism, racism – specifically anti-Black racism - patriarchy, sexism, fatphobia, and ableism to systematically exclude us from achieving our optimum wealth and health status, expose us to harmful toxins, and directly alter our anatomies over multiple generations, producing the condition of weathering via chronic toxic stress and trauma. Scholars have long worked to unmask the mechanisms behind this harmful narrative. Dr. Arline T. Geronimus coined the term “weathering” to describe the effects of oppression and exploitation —including racism and classism—on the body, especially the Black woman body, resulting in the acquisition of disease at younger ages, of higher severity, and experiencing shorter lifespans [8]. Furthermore, Christina Sharpe introduces the concepts of “weather” and “wake” from *In The Wake: On Blackness and Bein*g to describe the persistent and pervasive impact of anti-Blackness and the afterlives of slavery on Black life [9]. While “weather” represents the constant, pervasive conditions of anti-Black racism that surround and shape Black existence, akin to an inescapable, oppressive atmosphere, “wake” refers to the lingering effects of slavery, both as the lasting impact of the Middle Passage and as the ongoing presence of racial violence, shaping how Black people live and resist in the world today.
2. This **storyline** conceals how these dehumanizing systemic forces encapsulated in enslavement [10], slave patrols [11], Jim Crow segregation [12], de facto segregation [13], lynching [14], Redlining [15,16], Tulsa massacre [17], Eugenics, genocide [10] - directly exclude us from participating fully in our own humanity and in the society we built through stolen labor on stolen land, so that our access to quality affordable healthcare, nutritious food, childcare and education, housing, transportation, well-paying jobs, clean air, water, and soil, fair criminal justice experiences, climate responsive and resilient neighborhoods and communities, is severely restricted resulting in disparate morbidity, disability, and mortality.
3. This **storyline** masks the history of Black women as the reproductive incubator for the American chattel slavery enterprise, our bodies forcefully turned into factories of labor production, an economic powerhouse - birthing, nursing, and performing the work that built an empire. Those same bodies, while violated by both white men and women, were also forced to preserve the lives and legacy of their oppressors by breastfeeding and raising white babies as wet nurses, nannies, and mammies [18,19].
4. This **storyline** erases the role of organized medicine, particularly, obstetrics and gynecology in actively destroying the system of Black, Indigenous, and immigrant midwives who were the primary form of the full spectrum of reproductive healthcare in the US through the 19^th^ century and demonstrated outstanding maternal health outcomes. Or of physicians and scientists like Marion Sims who built their careers and produced advancements in medicine and science on the bodies, uteruses, and cervixes of Black women. Women such as Anarcha, Betsey, and Lucy the enslaved African women who endured painful surgical experiments without anesthesia by Marion Sims, a man commonly referred to as the so-called father of OBGYN; and Henrietta Lacks, the woman whose cervical cells, HeLa cells - the first immortal cervical cell line - were harvested without consent and used to create the most significant scientific innovations of our time such as the COVID19 and polio vaccines, cancer and HIV treatments, and space research [20].
5. Ultimately, this ahistorical a-contextual disrespectful **storyline** of ignoring, obscuring, concealing, and erasing the very active and present history of oppression, violence, and exploitation is in service of fulfilling the purpose of perpetuating the white supremacist delusional lie of inherent Black inferiority and sub-humanity. Thus, the blame for health disparities and poor outcomes can be laid squarely at the feet of the oppressed, while oppressive institutions, systems, and structures continue with impunity and in perpetuity, maintained by tons and tons and tons of resources. Millions and Billions and Trillions of dollars.

## A gap in the current literature

These narratives uphold, maintain, and reinforce systems of white colonial patriarchal classist ableist capitalism and are, therefore, agents of oppression upon the very same peoples they claim to represent. Existing literature on Black maternal health has begun to incorporate narrative approaches to challenge these dehumanizing storylines. Scholars have used storytelling, testimonio, and Black feminist autoethnography to rehumanize Black birthing experiences and contextual systemic harm. For instance, Dana-Ain Davis uses birth stories and ethnographic interviews in *Reproductive Injustice* to illuminate how Black women’s birthing experiences are shaped by medical racism and obstetric violence, emphasizing that poor outcomes are not the result of individual behaviors but of systemic neglect and surveillance [21]. Similarly, Kimberly Seals Allers and many others have advocated for the inclusion of maternal narratives to shift the gaze from deficit-based models to strengths-based storytelling rooted in lived experience and community resilience [22]. Scholars like LeConté Dill (p. 229–245) and Toni Bond (p.207-216) have utilized testimonio and participatory research methods to center the voices of Black birthing people as a form of resistance and reclamation [23].

These approaches center lived experiences and positionality, shifting away from purely quantitative public health data. However, many of these narratives remain tethered to trauma and survival when the counteracting force needed to dismantle these storylines is a *Vitality Framework* reliant on the presence, creation, and celebration of Black joy as a form of resilience, repair, healing, transformation, and liberation.

The Vitality Framework offers a distinct contribution by centering joy, imagination, and liberation as necessary components of health. Rather than simply resisting dehumanization, it affirms Black life as inherently worthy, abundant, and generative. This shift from “surviving harm” to “cultivating thriving” is not widely present in the current literature, and our framework responds to this by providing an alternative lens—one that reclaims the power of Black birthing experiences not only as sites of resistance, but also as sources of renewal, community, and future-building.

## Reframing Black health narratives by embracing Black Joy within a Vitality Framework

Black Joy exist in music, song, dance, worship, poetry, art, community, play, rest, work, fellowship, relationships, family, friendships, parenting, caregiving, teaching, serving, loving, and simply living. Anchored in early spirituals, which themselves are rooted in the spirituals sung *hundreds and hundreds* of years prior, the music we create today - Jazz, the Blues, Rock and roll, R&B, gospel, country, pop, hip hop, reggae, calypso, samba, maracatu, Bomba, Soukouss, Afrobeats, Amapiano, and so much more - serves as vehicles of joy. A joy that opens the door to our collective imagination, enabling us to create our collective consciousness what liberation actually looks like, sounds like, feels like, tastes like, IS like. A joy that activates our collective power to actualize that liberation in real life.

Modern day freedom songs from the 19th through the 21st century find their roots in these spirituals as they upheld movements such as the civil rights movements of the 1950s and 60s and even liberation movements in Russia, Eastern Europe, China, and South Africa. So, that whether we are being encouraged that “We shall overcome, we shall overcome, we shall overcome, someday” or instructed to “Emancipate yourself from mental slavery, none but ourselves can free our mind” [24], or to keep in mind that “You can get the money, you can get the power. But keep your eyes on the Final hour” [25]. We learn from our African forbears that to sustain the work of liberation is to be able to hold polarity, to hold the both-and the joy we **create** enables us to do just that.

It is that same Black joy which, as **Brandy Factory,** Co-Founder of *Upset Homegirls*, states “affirms that I am not a victim. I am an agent of change. It rejects the idea that violence, … injustice, discrimination, prejudice, and dominance over others are normal and acceptable actions” [26].

As **Tracey Michae’l Lewis-Giggetts,** author of *Black Joy: Stories of Resistance, Resilience, and Restoration* states Joy is a “tool in our arsenal, one that not only deeply disturbs the racist systems we are trying to dismantle but also offers a direct path to healing and wholeness as we do” [27].

Black joy is not, as **Kleaver Cruz** Founder of The Black Joy Project states, “… dismissing or creating an ‘alternate’ Black narrative that ignores the realities of our collective pain; rather, it is about holding the pain and injustice…in tension with the joy we experience. It’s about using that joy as an entry into understanding the oppressive forces we navigate through as a means to imagine and create a world free of them” [26].

Joy manifests differently for everyone and is known by various names, but at its core, it remains joy regardless of your walk of life, background, ethnicity, religion, or ability. Discovering what joy means to you is a deeply personal and reflective journey. This process is crucial for everyone, as it serves as the first step in freeing our minds from the imposed ways of thinking that have shaped us. And so, as we reflect on what Black Joy means to us, we are led down diverging but ultimately converging paths.

I, Ijeoma Opara am liberated from the tyranny of dichotomy as I learned that my grief did not preclude joy. My sorrow did not preclude joy. My anger, my rage, my pain, my fear, my uncertainty, my trauma, my doubts, do not preclude joy. Because my humanity does not preclude joy. Joy being the access point to the fullness of my humanity and therefore containing ALLLLL of the human things including grief.

I, Yasmine Elmi, am liberated as I recognize that my joy is not bound by the limitations imposed by others or myself. I fully embrace the totality of what my joy means rejecting the notion that it needs to take on a specific form. It means understanding that my capacity for joy is boundless, and that every moment of happiness is a testament to my resilience and strength.

This is only the beginning of what it means to truly be free and to embody liberation, unlocking an abundant thriving vitality. We assert that Black joy invites us to reject that storyline by beginning to reimagine this narrative as its rightful owners, to reframe it as a true representation of Black maternity that transcends mere morbidity and poor health outcomes, and to reclaim and recreate it by pouring life, joy, and vitality back into our storylines and thus ourselves reimagine, reframe, reclaim, and recreate the narrative of maternal health as well as health and wellbeing in general. It calls for us to flip the script, expand the scope, and complete the story. Black joy gives us a language of life, love, and liberation so that instead of Black maternal mortality we begin to speak about Black maternal VITALITY.

## The Vitality Framework: Centering Black Joy and Liberation

**Fig 1:**
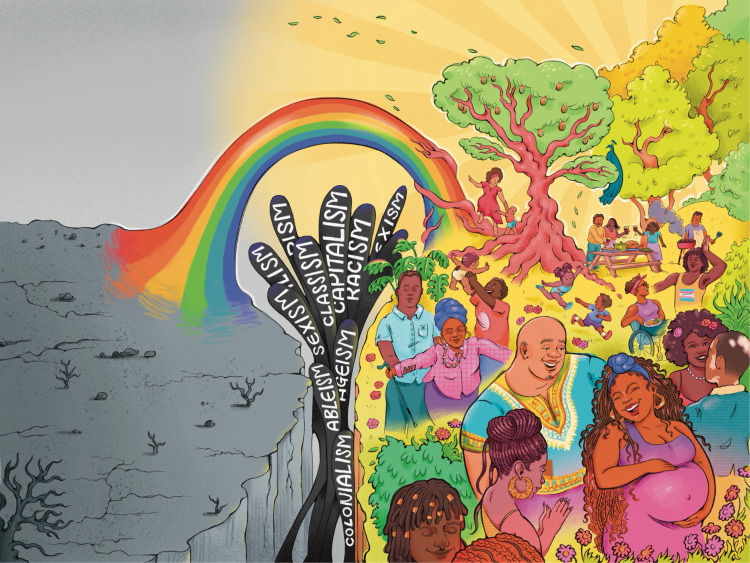
Vitality Framework: A peacock symbolizing the Vitality Framework, with feathers representing Reimagine, Reframe, Reclaim, and Recreate, leading to Joy, Liberation, and Vitality. Republished from Dr. Ijeoma Nnodim Opara under a CC BY license, with permission from Sceyence Studios, original copyright 2025.

The *Vitality Framework* (Fig 1) represents a transformative approach to understanding and addressing Black maternal health, emphasizing not just survival but living, not just life but vitality. It challenges the ‘dominant narratives’ in Black maternal health scholarship and media coverage that focus solely on morbidity and mortality, on crisis and deficiency. This ‘dominant narrative’ emphasizes racial disparities but often fails to contextualize them within broader structures of racial capitalism, medical racism, and reproductive justice [8]. This proposed presents a holistic view that celebrates the full spectrum of Black lived experiences. This framework is inspired by the concept of Black joy, recognizing its power as a source of resilience, healing, and liberation.

### Reimagine

A *Vitality Framework* does not mean we ignore the mortality, disability, morbidity reports, rather it provides an expanded, empowered, and complete lens by introducing a critical historical socio-political context that asks different questions and leads to different solutions – solutions generated by Black women and birthing people, for Black women and birthing people, benefitting...frankly, everyone. This *Vitality Framework* invites us to listen to Black women’s stories about what motherhood means to them, encompassing the many ups and upsets, triumphs and tribulations, celebrations, and crises. It prompts us to ask Black women what they envision, desire, and are striving for in terms of their health and wellbeing.

### Reframe

Through a vitality lens, We SEE them, BELIEVE them, VALUE them, INVEST in them, and FOLLOW them as they drive the momnibus of change to flip weathering into “Ethering”. We define this as a reversal of the erosive effects of exposure to the exploitation, oppression, violence of racism and sexism on our bodies so that, instead, there is an accretion or a buildup of protective transcendent triumphant features constituting a melanated vitality in response to and despite the trauma, oppression, and violence of white supremacism, and anti-Blackness.

### Reclaim

**Fig 2:**
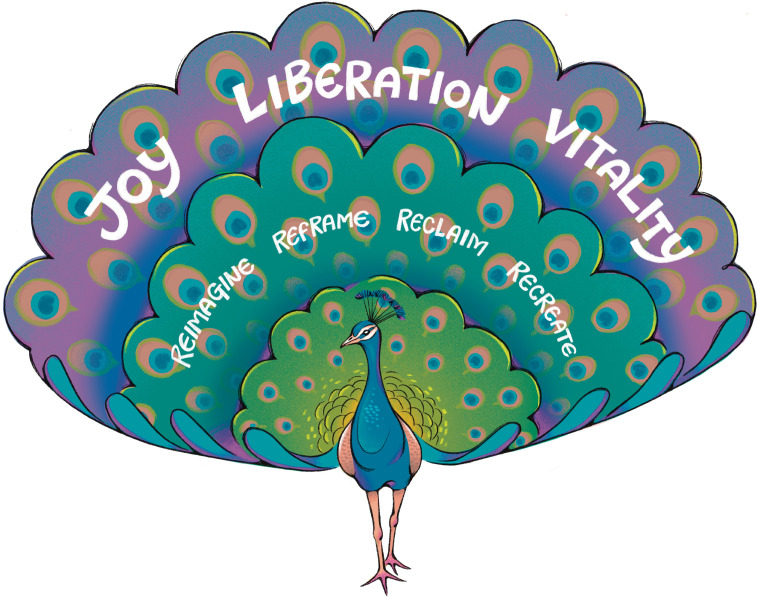
Ethering as A Path from Weathering to Vitality: A visual contrast between weathering (a barren landscape symbolizing oppression) and ethering (a thriving, vibrant community), connected by a rainbow bridge representing transformative healing. Republished from Dr. Ijeoma Nnodim Opara under a CC BY license, with permission from Sceyence Studios, original copyright 2025.

**Misogynoir,** as coined by Dr. Moya Bailey, is the combination of racism and sexism – defined as a hatred of, aversion to, and prejudice towards specifically Black women. But when we are **ethered**, we turn misogynoir into *joie de femme Noire* (Black woman joy) that defies societies attempts to define us, demean us, dehumanize us **thereby reclaiming and recreating our storyline at once** (Fig 2).

It is that *joie de femme Noire* that allows us to define ourselves, give meaning to our lives, honor our humanity, and create our destinies. *Joie de femme Noire* was at work when Black women, the women of African Descent for Reproductive Justice collective, created the Reproductive Justice movement in 1994 in Chicago [28]. They recognized that the women’s rights and reproductive rights movement, dominated by white middle class and wealthy women, would not and could not represent them. As a level setter, Reproductive Justice is the complete physical, mental, spiritual, political, social, and economic well-being of women and girls, based on the full achievement and protection of women’s human rights [29]. It fights for the right to have a child; the right not to have a child; and the right to parent the children we have, as well as to control our birthing options, such as midwifery. It also fights for the necessary enabling conditions to realize these rights and so it is comprehensive, holistic and intersectional, because as Audre Lorde told us, “There is no such thing as a single-issue struggle because we do not live single-issue lives” [30]. It concerns itself with the fight for queer people, for trans people, for people with disabilities, for immigrants, for the poor; because none of us is free until all of us are free as we recognize that heteronormativity and heterosexism, ableism, xenophobia, classism are all fruit of the poisoned white patriarchal supremacist capitalist tree.

### Recreate

When we are **ethered**, this *joie de femme Noire* becomes a living and breathing extension of Ubuntu, an ancient and powerful African word meaning *I am because we*. Our liberation and existence are intertwined, but when we are ethered, so is our joy. For Madiba Nelson Mandela, Ubuntu represents “the profound sense that we are human only through the humanity of others; that if we are to accomplish anything in this world, it will in equal measure be due to the work and achievements of others” [31]. In this same manner, the process of ethering and exploring a *Vitality Framework* is both and individual and communal journey. It will look different for everyone, but if we are to achieve, experience, and become *joie de femme Noire,* it will be thanks to the collective and individual efforts of us all. Our joy is inextricably linked.

When we are **ethered**, the transcendental power of *joie de femme Noire* manifests as it did in 2023 when the state of Michigan enshrined the right to an abortion into the state constitution, in response to the US supreme court’s ruling that ended a woman or birthing person’s right to an abortion. This magnificent Michigan feat was only made possible by the tireless advocacy and activism of a Black woman-led multi-ethnic reproductive justice movement rooted in the living bedrock of those founding mothers almost 30 years prior. *Joie de femme Noire* powers and sustains the ongoing work to pass the Black maternal health momnibus act into law. A result of the courageous, innovative activism of organizations such as Black Mamas Matter Alliance, Black Mothers Breastfeeding Association, & Black Women Birthing Justice, Inc, amongst others, the Momnibus act, sponsored by representative Lauren Underwood and Senator Cory Booker, is a comprehensive set of 13 individual bills that will, among other things, make critical investments in social determinants of health that influence maternal health outcomes, like housing, transportation, and nutrition; as well as Invest in community-based initiatives to reduce levels of and exposure to climate change-related risks for moms and babies. A container for the 3 tenets of effective movement building – service, advocacy, and organizing – *Joie de femme Noire* makes it possible for Black women led Birthing practices like Ubuntu Black Family wellness collective led by Dr. Michelle Drew, or Mama Michelle, who is a certified midwife and family nurse practitioner, to boast of incredible birthing outcomes far outpacing the state and national statistics [32]. These organizations are in the business of in **Black maternal VITALITY**. They demonstrate that community based culturally congruent midwifery led care is vital to achieving a liberated world where Black women and birthing people thrive, and health equity is the norm.

When we are **ethered**, we embrace rest as our human right. Again, Audre Lorde not only reminds us that Joy is an act of resistance and that we don’t live single issue lives but that Caring for ourselves is not self-indulgence, it is self-preservation, and that is an act of political warfare [33]. Black joy mandates self-love, self-care, and self-compassion. Dr. Tricia Hersey, The nap bishop of the Nap ministry and author of the book Rest is Resistance: A manifesto states “You were not just born to center your entire existence on work and labor [34]. You were born to heal, to grow, to be of service to yourself and community, to practice, to experiment, to create, to have space, to dream, and to connect.” and she goes on to say, “Loving ourselves and each other deepens our disruption of the dominant systems.” Black joy is how we love ourselves and each other.

When we are **ethered**, our vitality transcends time as our joy enables us to ground ourselves in history, which is ongoing, grapple with the present, which is uncertain and challenging, and access a future that sings, dances, and celebrates our continued existence, thriving, and abundance. Black history is every day, and every day is Black history, present, and future.

Black joy imagines a future that is already here and boldly engages our own solutions to problems labeled as “wicked” only because the people most impacted are excluded from the decision-making tables. Through an Afrofuturistic lens which not only sees us IN the future but also sees us WIN the future, we innovate resolutions and demand accountability for the issues related to climate change, ecosystem & biodiversity health, animal health, planetary health, environmental justice, data and technology (AI), foodways, education, transportation, the economy, neighborhoods, Gaza, Rafah, Palestine, Congo, Myanmar, Sudan, and everywhere there is injustice, human rights violations, war crimes...genocide.

## Implications of Joyful systems for Policy, Research and Practice with

When we are **ethered**, Black joy leverages our collective imagination to create **joyful systems** led by the most multiply marginalized that disrupt and eliminate the ongoing active presence of oppressive exploitative violent structures of law, policy, rules, and regulations. Unjust structures that produce the practices, behaviors, norms, and culture resulting in observed inequities. Joyful classrooms, offices, schools, departments, institutions, universities, organizations, clinics, hospitals, health centers, health departments, neighborhoods, communities, cities, states, provinces, parishes. Nations. What could it look like to be a nation of joy, in spite of, and maybe even because of, our challenges? What becomes possible when we center joy in our self-care and compassion, our interactions, relationships, work, parenting, research, practice, studies, and politics?

Could we create vitality agents and agencies of joy that create liberation communities which foster nurturing, softness, and safety where everyone feels valued and valuable, particularly those from the most multiply minoritized communities? Communities where everyone is inspired and rewarded for showing up authentically, passionately and purposefully and supported to achieve their highest potential for health, wealth, and happiness?

When we are **ethered** we ask - Where could each of us adopt a *Vitality Framework* and inject some Black joy to facilitate the ethering of our bodies versus the weathering? How could we lean into possibility and unleash our imaginations to weave in *joie Noire* into a course, an activity, a meeting, a dinner?

You may doubt your ability for revolution, but as the African proverb goes, “if you ever think you’re too small to have a big impact, you’ve never spent the night with a mosquito”. Similarly, the anthropologist Margaret Mead reminds us, “Never doubt that a small group of thoughtful, committed individuals can change the world. In fact, it’s the only thing that ever has” [35]. Building on this, a small group of thoughtful committed organized, strategic, joyful individuals can create a significant change. The injustices of the world are all interwoven into a wretched fabric of loose threads, but as we inhabit, embody, and deploy joy within our various spheres of influence we tug on these threads gradually unravelling the whole material. This collective effort, grounded in joy and vitality can disrupt and dismantle the structures that perpetuate any inequity and injustice.
