# Correction: Reimagining Black maternal health narratives: Embracing a Vitality Framework for joy, liberation, and healing

**DOI:** 10.1371/journal.pgph.0005361

**Published:** 2025-10-21

**Authors:** Ijeoma Nnodim Opara, Yasmine M. Elmi

The images for Figs 1 and 2 are incorrectly switched. The image that appears as Fig 1 should be Fig 2, and the image that appears as Fig 2 should be Fig 1. The figure captions appear in the correct order. The authors have provided a corrected version of figures here.

**Fig 1:**
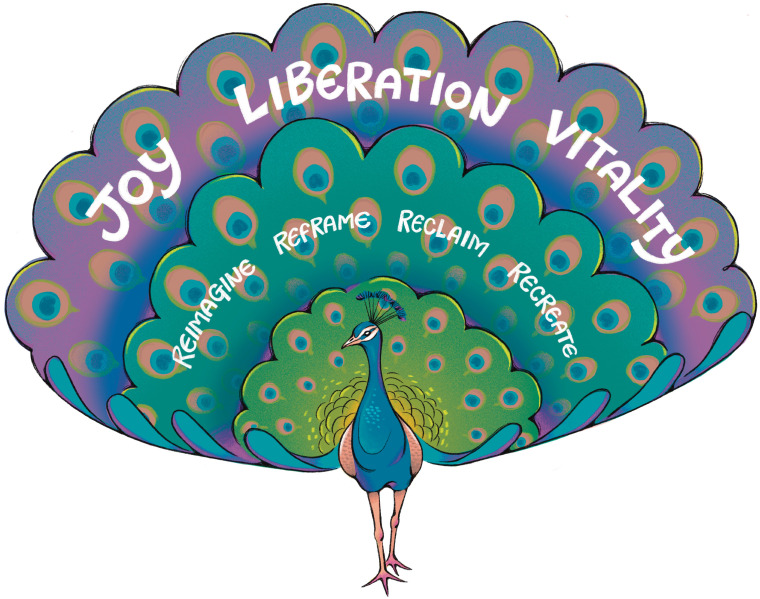
Vitality framework: A peacock symbolizing the vitality framework, with feathers representing reimagine, reframe, reclaim, and recreate, leading to joy, liberation, and vitality. Republished from Dr. Ijeoma Nnodim Opara under a CC BY license, with permission from Sceyence Studios, original copyright 2025.

**Fig 2:**
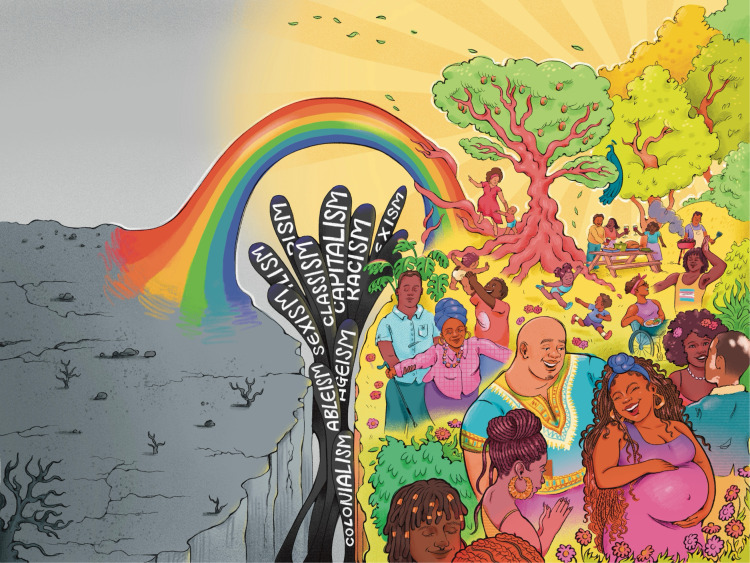
Ethering as a path from weathering to vitality: A visual contrast between weathering (a barren landscape symbolizing oppression) and ethering (a thriving, vibrant community), connected by a rainbow bridge representing transformative healing. Republished from Dr. Ijeoma Nnodim Opara under a CC BY license, with permission from Sceyence Studios, original copyright 2025.
